# Influence of Water, Sanitation, and Hygiene Practices on Common Infections among Under-Five Children in Longido and Monduli Districts of Arusha, Tanzania

**DOI:** 10.1155/2017/9235168

**Published:** 2017-09-25

**Authors:** Hoyce Amini Mshida, Neema Kassim, Martin Epafras Kimanya, Emmanuel Mpolya

**Affiliations:** ^1^School of Life Sciences and Bioengineering, Nelson Mandela African Institution of Science and Technology (NM-AIST), P.O. Box. 447, Tengeru, Arusha, Tanzania; ^2^Department of Food Biotechnology and Nutritional Sciences (FBNS), School of Life Sciences and Bioengineering (LiSBE), Nelson Mandela African Institution of Science and Technology (NM-AIST), P.O. Box. 447, Tengeru, Arusha, Tanzania; ^3^Department of Rural Economy and Agriculture, African Union Commission, P.O. Box 3443, Addis Ababa, Ethiopia; ^4^Department of Global Health and Biomedical Sciences, School of Life Sciences and Bioengineering (LiSBE), Nelson Mandela African Institution of Science and Technology (NM-AIST), P.O. Box 447, Arusha, Tanzania

## Abstract

The study aimed at assessing water, sanitation, and hygiene practices and their influence on infectious diseases among under-five children in semipastoral communities of Arusha. The study was cross-sectional in design. Prevalence of infectious diseases among under-five children was derived from patients' attendance register. Mothers randomly sampled from households were interviewed using questionnaire. Information regarding child morbidity and sociodemographic and WASH characteristics was gathered. Hospital data revealed that 2/3 of under-five patients visited the hospitals annually were suffering from infectious diseases. Mean percentage of diarrhea prevalence for years 2013–2015 in Longido was higher than the mean of the respective years prevalence in Monduli (*p* = 0.02). Households' survey showed that 15.5% of under-five children were suffering from diarrhea. Children who consumed foods kept in* kibuyu* (*p* < 0.001) or used unboiled cows' milk (*p* = 0.01) or were drinking surface water (*p* = 0.04) or born to uneducated mothers (*p* = 0.01) had increased risk of developing diarrhea compared to their counterparts. Storing complementary foods in* kibuyu* was strongly associated with diarrhea among under-five children. To address the problem, communities under study need to be motivated through health education on food hygiene, proper handling of food storage containers, and domestic water treatment at the household level.

## 1. Introduction

Diarrhea remains the second leading cause of mortality and morbidity among under-five children worldwide and the first in sub-Sahara Africa (SSA) [1, 2]. Despite the fact that diarrhea can be cured and prevented, it still kills about 1.26 million young children annually worldwide which exceeds the mortality due to malaria, tuberculosis, and HIV/AIDs combined [3]. Furthermore, the lives of about 800 under-five children are lost daily due to diarrhea worldwide [4] and the highest rates of child mortality are in SSA and Asia [2, 5, 6].

A number of studies [7–12] have associated the high prevalence of diarrhea in developing countries with poor WASH practices. Soil-transmitted infections such as Ascariasis, whipworms, and hookworms that affect about 1.2 billion, 800 million, and 750 million people worldwide, respectively, have also been associated with poor WASH practices [13]. Similarly, environmental enteropathy which is mainly a result of regular ingestion of fecal bacteria due to poor sanitation and hygiene conditions has been reported in under-five children [14, 15]. Factors such as unhygienic handling and storage of foods, poor handwashing practices, poor disposal of child feces, open defecation, lack of safe water sources for domestic purposes by the majority, and poor solid and liquid waste disposal continue to be major health threats among under-five children. Other factors include socioeconomic conditions of the household such as literacy, level of income of the family members, and early introduction of CF to children which may increase chances of water and food-borne diseases [1, 12, 16, 17]. However, improved WASH practices may significantly minimize the incidences of WASH-associated infections among under-five children [18–21].

Tanzania is among the East and Southern African countries that did not meet the Millennium development goals 3 and 7 target 7C to halve by 2015 child mortality and proportion of people without sustainable access to water supply and basic sanitation [19, 22, 23]. This failure could be due to population growth characterized by rapid urbanization, which the Government is unable to serve due to limited capacity and resources. Even though Tanzania has implemented a number of national campaigns including* Mtu ni Afya* (To be Healthy is to be Human),* Maji ni Uhai* (Water is life), and the national sanitation and hygiene campaigns as a way of improving WASH practices and reducing WASH-associated infections [24–26], yet about 14% of its households still practice open defecation, 76.4% use unimproved pit latrines, and 42.7% use unsafe water sources such as rivers and ponds for domestic purposes [23, 24, 27]. Furthermore, about 9% of deaths among under-five children are due to diarrhea [16, 27] and about 12% of under-five children suffer from diarrhea associated infections, annually [28].

WASH-associated infections among under-five children are endemic in semipastoral communities as per reviewed hospital records of 2013 to 2015. Also, several studies have reported a strong correlation between WASH practices and infectious diseases among under-five children. It is further known that WASH practices among semipastoral communities are poor but the extent such practices influence infectious diseases has not been documented. This study, therefore, intended to assess the influence of WASH practices on infectious diseases particularly diarrhea associated infections among under-five children in semipastoral communities of Longido and Monduli Districts of Arusha. It is expected that findings from this study will inform policy makers about programmatic interventions that have potential to stem the problem in the population under study as well as add knowledge to the world of science.

## 2. Materials and Methods

### 2.1. Study Area

The study was conducted in semipastoral communities of Longido and Monduli Districts of Arusha region, in the North Eastern corner of Tanzania. The districts are bordered with Kenya to the North, Kilimanjaro region to the East, Manyara region to the South, and Simiyu and Mara regions to the West. Monduli district covers 6,992.67 km^2^ and has a population of 158, 929 people and is divided into twenty wards. Longido district covers 7,885.01 km^2^ and has a population of 123,153 people and is divided into eighteen wards. The Maasai tribe is the dominant ethnic group in both districts and majorities are seminomadic in nature. Other tribes include Mang'ati and Waarusha.

### 2.2. Study Design and Tools

The research employed the cross-sectional study design in which a standardized questionnaire adopted from the UNICEF survey on monitoring WASH practices at household level in Gaza in 2009 was modified to fit the semipastoral communities and administered to mothers/caregivers. Information on WASH, sociodemography, morbidity, and child feeding practices were gathered. Hospital records from the respective district hospitals were reviewed and trends of common infections among under-five children who attended the hospitals for three consecutive years (2013 to 2015) were derived.

### 2.3. Sampling and Sample Size

A multistage sampling technique was employed whereby Longido and Monduli Districts were purposively selected for this study. Simple random sampling technique was employed to obtain four wards, two from each district. One village from each ward was randomly selected whereby Orbomba and Kimokouwa villages from Longido and Meserani and Makuyuni villages from Monduli were sampled. From each village, households with under-five children were purposively selected for the study. For households with more than one under-five children, only one child was randomly selected for the study. Sample size was calculated based on the cross-sectional study sample size calculation formula as follows: *n* = *z*^2^*P*(100 − *P*)/*ε*^2^

*n* is a minimum sample size; *z* is a value corresponding to the confidence level = 1.96; *P* is anticipated prevalence 25% of WASH practices among semipastoral communities; and *ε* is a margin of error which is 5% with confidence interval of 95%. A total of 310 mother-child pairs, 150 from Longido and 160 from Monduli, were sampled. The sample size took into consideration 7.2% attrition rate.

### 2.4. Ethical Clearance

Ethical clearance was obtained from National Institute for Medical Research (NIMR) of Tanzania. Informed written consent was sought from mothers and caregivers of the children under study prior to administration of the questionnaire. Confidentiality regarding the information collected from the survey was ensured.

### 2.5. Inclusion and Exclusion Criteria

Children aged 6–59 months were involved in this study. Children who were seriously ill or under medication during survey were excluded from the study.

### 2.6. Definition of Terms

Diarrhea in this study was regarded as a common symptom of gastrointestinal infections caused by pathogens such as bacteria, viruses, or protozoa and characterized by child having loose or watery stools at least three times per day or more frequently than normal [29]. WASH-associated infections were referred to as water and food-borne infections that are normally transmitted through fecal-oral routes such as diarrhea diseases and Helminths [12]. Semipastoral communities were referred to as communities engaged more on raising livestock and partly cultivating food crops in small scale and have a tendency of moving from one place to another to search for fresh pastures and water for their herds especially during dry season [30, 31]. Maasai communities are a good example of semipastoral communities and are the ones studied in this study.

### 2.7. Data Management and Analysis

Data collection tools were pretested in semipastoralists communities of Arusha district and validated accordingly. The household survey was conducted by well trained and qualified enumerators. Data cleaning was done every evening after survey and then double entered into Epi-data version 3.3.1 by two different qualified personnel before being transferred into SPSS Version 20 for statistical analysis. Descriptive analysis was done to obtain frequencies and percentages of WASH variables, child feeding practices, child morbidity information, and sociodemographic characteristics of the study subjects. Logistic regression analysis was done to look for variables associated with diarrhea. Variables with* p* value less than 0.05 were then run through multivariable model using backward Wald method and confounders such as vaccination against rotavirus were controlled. Variables with* p* values less than 0.05 were considered as independent predictors for diarrhea associated infections among under-five children. Again independent sample* t*-test was done; hospital data and mean percentages of WASH-associated infections for both Monduli and Longido district hospitals were compared.

## 3. Results 

### 3.1. Common Infections among Under-Five Children

Data reviewed from Longido and Monduli district hospitals revealed that about two third of the under-five patients visited the district hospitals seeking medical care were suffering from infectious diseases. Diarrhea associated infections were regarded as proxy for WASH-associated infections. On average, WASH-associated infections among children under-five years of age as obtained from Longido hospital attendance register for years 2013–2015 were diarrhea associated infections (13.4%,) and intestinal worm infections (3%) and for Monduli district hospital were diarrhea associated infections (10%) and intestinal worm infections (3%). Independent* t*-test analysis did not show any significant difference between mean percentages of overall infectious diseases in the two districts (*p* = 0.6). However, prevalence of diarrhea associated infections among under-five children residing in Longido was significantly higher compared to children from Monduli (*p* = 0.02) although for worms infestation it was not the case (*p* = 0.4).  Table 1 shows mean percentages of WASH-associated infections among under-five children that attended Longido and Monduli district hospitals for three consecutive years.

### 3.2. Sociodemographic Characteristics of the Study Participants and Variation of Diarrhea Prevalence

The study employed 310 pairs of mothers/caregivers and children aged 6–59 months. The mean age of the children was 29.8 ± 17.1 months. Mothers aged 20–29 years constituted the largest group (55.8%). About half of the mothers did not have any formal education, whereas the rest attained primary education (45%), secondary education (5%), and college/university levels of education (1%). Most (96.1%) of mothers were married and more than half had more than one under-five year's old children. The number of under-five children per mother ranged from one to four. Data from questionnaire also revealed that majority of under-five children in Longido were suffering from WASH-associated infections such as diarrhea associated infections (16.9%). In Monduli, the prevalence of WASH-associated diseases such as diarrhea is 13.3%.  Table 2 shows the distribution of prevalence of diarrhea within the period of two weeks prior survey date in relation to sociodemographic characteristics of the study subject.

### 3.3. WASH Practices

#### 3.3.1. Domestic Water Sources

**Figure 1:**
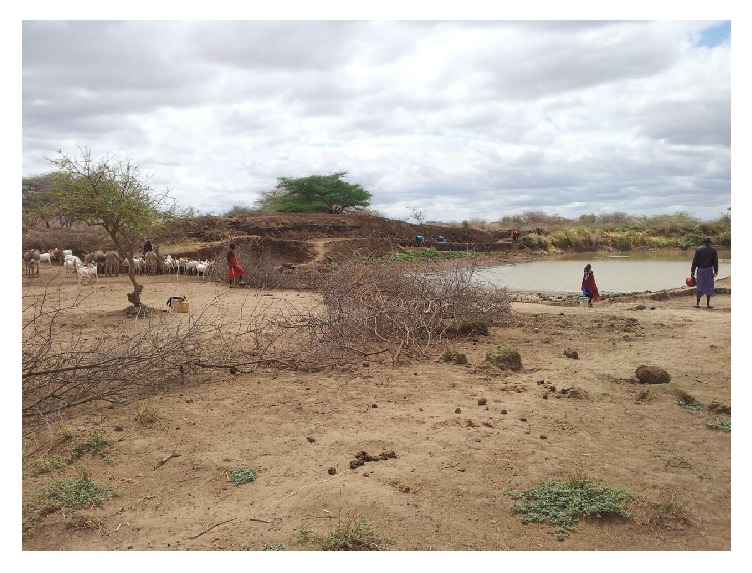
Surface water from pond/*silange,* a main source of domestic water for people from Orbomba village.

Respondents from both districts reported to obtain water from different sources on seasonal basis. About 85% and 100% of respondents from Longido and Monduli, respectively, reported to access less than 20 litres of water per person per day for general uses. About 35% of respondents from Longido were depending mainly on surface water for domestic purposes compared to 11.5% of respondents from Monduli.  Figure 1 shows the surface water pond traditionally known as* silange* which is mainly used as domestic water source by people from Orbomba village.

#### 3.3.2. Sanitation Practices

**Figure 2:**
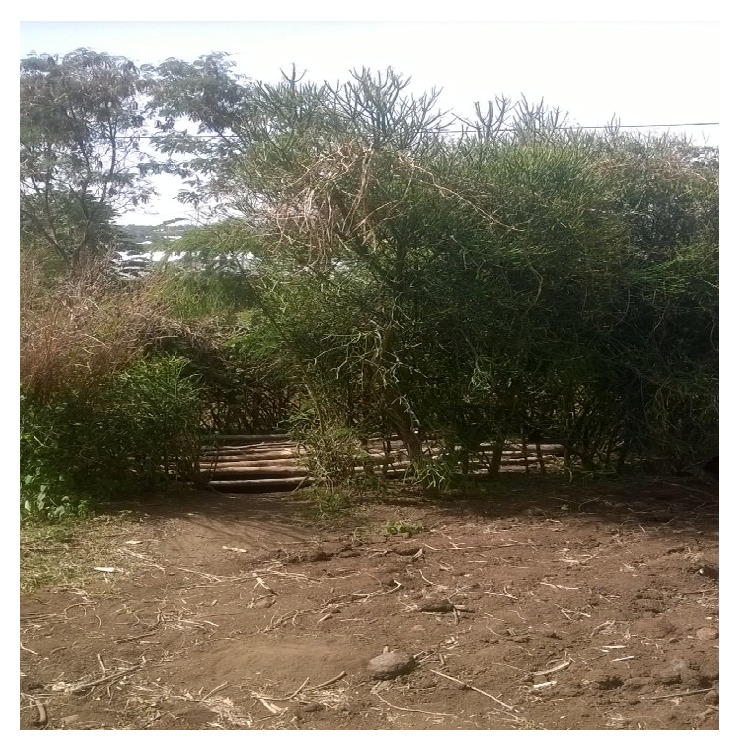
Traditional pit latrine from Kimokouwa village.

Results showed that about 21% and 6.3% of households from Longido and Monduli, respectively, had latrines of which two-thirds were traditional pit latrines (Figure 2). Open defecation was practiced by 44% and 55.6% households from Longido and Monduli, respectively, and the rest were practicing other defecation methods including cut style defecation method (digging small whole using a hoe to defecate in it and burry). Disposal of child feces to the toilets was practiced by only 20% of participants from Longido and 5.6% from Monduli. More than half of the respondents from both Longido and Monduli reported to use children feces in feeding their dogs with a belief that children feces are harmless and useful to dogs.

#### 3.3.3. Hygiene Practices

**Figure 3:**
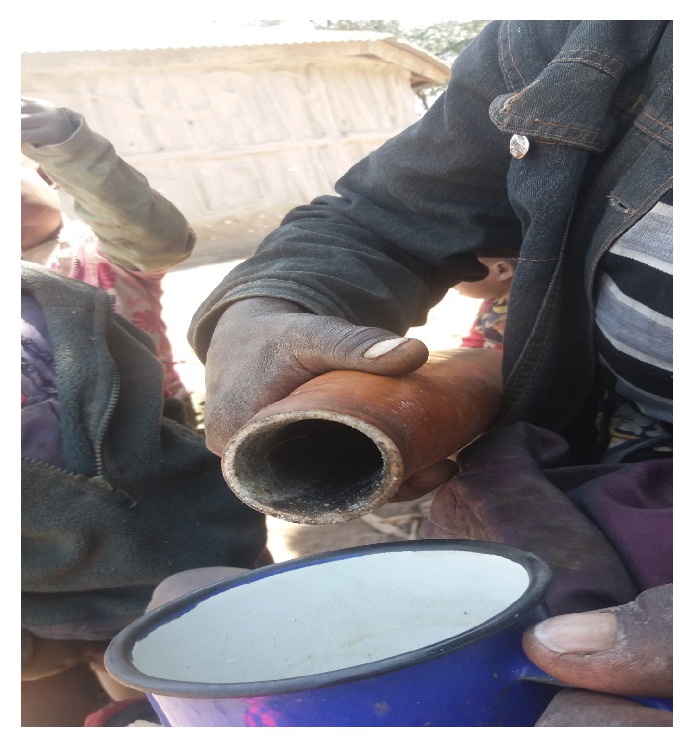
A Maasai woman pouring milk from* kibuyu* ready to feed her child.

Hand washing with soap during critical moments was practiced by 9.3% of respondents from Longido and 2% from Monduli. Complementary foods were kept in either Thermos, hotpot, or* kibuyu* after preparation (Figure 3). The latter case was being used by about 20% and 11.3% of participants from Longido and Monduli, respectively. In parallel, 78.4% of the respondents from Longido and Monduli Districts reported to boil cow's milk prior feeding. When they were asked to explain how boiling of cows' milk was done, it was noted that about half of them were just warming the milk and not boiling. Ten percent and 2% of participants from Longido and Monduli, respectively, reported to boil water for drinking purposes.

### 3.4. Predictors of Diarrhea

Diarrhea prevalence among under-five children in the population under study was predicted by environmental, behavioral, and sociodemographic factors. Children fed on CF kept in* kibuyu* were 4 times more likely to suffer from diarrhea compared to those fed on CF kept in Thermos or hotpots (AOR = 4.1; 95% CI = 4.3–19). Children feed on cow's milk which was not boiled were about 3 times more likely to develop diarrhea compared to those feed on boiled cow's milk (AOR = 2.9; 95% CI = 1.3–6.3). Mothers who reported using tap water for domestic purposes were more than one time less likely to have children suffering from diarrhea compared to those who reported using surface water such as ponds/*silange* (AOR = 1.2; 95% CI = 1.02–4.1). Children born to mothers with no formal education were 1.3 times more likely to develop diarrhea compared to children born to mothers with formal education (AOR = 1.3; 95% CI = 1.1–5.6). Children residing in Kimokouwa and Orbomba villages of Longido were 3 times more likely to develop diarrhea compared to children residing in Makuyuni villages of Monduli (OR = 3.2; 95% CI = (3.3–10.2)). There was no association between the practice of giving child feces to dogs and diarrhea among under-five children.  Table 3 illustrates independent predictors of diarrhea among children under-five years of age in semipastoral communities of Longido and Monduli Districts.

## 4. Discussion

This study documents WASH practices in semipastoral communities and their influence on WASH-associated infections particularly diarrhea. Storage of already prepared CF such as milk and porridge in* kibuyu* for later use is a common practice among semipastoral communities and revealed an association with diarrhea among under-five children. Food stored in* kibuyu *may be rendered unsafe to children due to the fact that* kibuyu* is rarely washed and rinsed with herbal extract which is traditionally believed to preserve foods. The usefulness of the herbal extract is not proven and its safety is not determined. In addition, the child sips food from the* kibuyu *directly and may introduce pathogens to the food. The findings from present study are in line with other studies which reported that poor handling and storage of CF may have a significant contribution to diarrhea among children [16, 32–34]. However, proper storage of already prepared CF through washing of food storage containers and safe clean drinking water may reduce risks of diarrhea among under-five children in the population under study.

Cow's milk is commonly used in African societies as CF for children due to the fact that the majority do not afford baby formulas. Findings from this study showed that children fed on cow's milk which is not boiled were at higher risk of developing diarrhea compared to those fed on boiled cows' milk. This could be due to the reason that cows' milk may be a vehicle for transmission of pathogens from the host animal or the milk might have been cross-contaminated during milking. The findings from the present study are similar to findings from other studies [32, 33, 35–38] which reported an association between diarrhea and feeding children unboiled cow's milk. In addition, it has been reported by other studies that about 15% to 70% of diarrhea among children under-five years of age worldwide could be reduced by ensuring hygienic handling, storage, and thorough cooking of CF [34, 39, 40].

In spite of the fact that surface water is unsafe for domestic purposes and may lead to waterborne diseases, it was noted that surface water is the main source of domestic water for majority of the population under study. Findings from this study confirmed that children from households with access to tap water had less odds of developing diarrhea compared to children from households using surface water for domestic purposes. Possible sources of surfaces water contamination in these communities could be open defecation, offering children feces to dogs and sharing water sources with livestock which were reported by the majority. Such unsanitary practices may increase chances of surface water contamination especially during rainy season. The findings are in line with findings from other studies [1, 10, 27, 41, 42] which reported high prevalence of diarrhea among children residing in households relying on surface water for domestic purposes. Ensuring safe water for domestic purposes, through water treatment at the point of use including use of water filters, may reduce incidences of diarrhea among under-five-year-old children in these communities. Boiling of water for drinking purposes could also minimize the problem of diarrhea although water may be recontaminated during cooling and also the practice may be economically and environmentally unsustainable to the communities under study.

Besides that, children born to mothers with no education were at higher risk of developing diarrhea compared to children belonging to educated mothers. The reason could be that educated mothers may be more exposed to child care aspects including safe preparation, handling and storage of food, proper disposal of child feces, and proper feeding practices. The findings from this study are in agreement with findings from other studies [17, 27, 43] which reported high prevalence of diarrhea among children born to mothers with no education.

Place of residence has also been identified as one of the factors contributing to incidences of diarrhea [10, 43]. Children from Longido were at higher risk of developing diarrhea compared to children from Monduli. This could be due to the fact that majority of respondents from Longido reported using surface water from ponds* (silange)* which may be unsafe compared to respondents from Monduli district who had access to tap water emanating from military piped water source. The other reason could be storage of CF into kibuyu which was a potential risk of diarrhea and it was practiced more by Longido communities than Monduli. Findings from other studies [23, 44, 45] also reported place of residence as one of the factors associated with diarrhea.

## 5. Conclusion

Prevalence of WASH-associated infections particularly diarrhea associated infections among semipastoral communities of Arusha was higher compared to that of National Demographic and Health Survey of 2015 and could be contributed by poor WASH practices. Children fed on CF kept in* kibuyu* or cow's milk which was not boiled or belong to uneducated mothers or residing in Longido had higher risk of developing diarrhea compared to their counterparts. To address the problem under study multidisciplinary strategies targeting on health education including food hygiene and safety and treatment of drinking water along value chain may reduce the prevalence of diarrhea associated infections among under-five children in the study population. Also, studies to examine the magnitude of CF contamination particularly food kept in* kibuyu* as well as impact of diarrhea associated infections on nutritional status of under-five children are recommended to further address the problem of child mortality in the population under study.

## Figures and Tables

**Table 1:** Mean percentages of infectious diseases among under-five children for three consecutive years (2013 to 2015) as per Monduli and Longido district hospitals attendance.

Type of disease	Mean %		*p* value
	Longido	Monduli	
Infectious diseases	70.9 ± 5.3	72.8 ± 1.7	0.6
WASH associated infections			
Diarrhea associated infections	13.4 ± 1.3	9.8 ± 1.1	0.02
Worm infestation	2.9 ± 0.6	3.3 ± 0.3	0.4

**Table 2:** Variation of diarrhea prevalence among under-five children in relation to sociodemographic characteristics.

Variable	*n *	Diarrhea %	*p* value
Sex of the child			
Male	155	27.1	
Female	155	29.7	0.6
Age of the child			
6 months–11 months	58	29.3	
12 months–23 months	64	31.2	0.8
24 months–59 months	188	27.1	0.7
Time the CF was introduced to the child			
Before 6 months	278	27.7	0.4
6 months and above	32	34.4	
Breast feeding status			
Yes	119	24.1	
No	191	35.3	0.03
Education status of the mother			
No education	145	31.7	0.2
With education	165	25.5	
Age of the mother			
20–29 years	173	32.4	0.6
30–39 years	98	25.5	0.9
40 years and above	30	13.3	
Village			
Makuyuni	81	12.3	
Meserani	79	12.7	0.7
Kimokouwa	64	43.1	0.001
Orbomba	86	43.7	0.001

CF, complementary foods. Diarrhea prevalence was higher in Kimokouwa and Orbomba villages of Longido district compared to Makuyuni and Meserani of Monduli district.

**Table 3:** Predictors of diarrhea among children under-five years of age.

Variable	*n*	Diarrhea %	COR (95% CI)	*p* value	AOR (95% CI)	*p* value
Education status of the mother						
No education	145	31.7	1.4 (0.9–2.2)	0.2	1.3 (1.1–5.6)	0.01^*∗*^
With education	165	25.5	1		1	
Age of the mother						
20–29 years	173	32.4	1.4 (0.3–6.2)	0.6	0.2 (0.1–0.8)	0.02
30–39 years	98	25.5	1.1 (0.3–4.3)	0.9	0.3 (0.1–1.4)	0.1
≥40 years	30	13.3	1		1	
Preparation of cow's milk prior to feeding the child						
Boiled	243	19.3	1		1	
Not boiled	67	61.2	6.6 (3.7–11.8)	0.001	2.9 (1.3–6.3)	0.01^*∗*^
Storage of prepared complementary food						
Thermos/hotpot	213	13.1	1		1	
Calabash	97	62	4.7 (6.1–18.9)	0.001	4.1 (4.3–19)	0.001^*∗*^
Source of drinking water						
Tap water	166	17	1		1	
Surface water	144	41.7	3.5 (2.1–5.9)	0.001	1.2 (1.02–4.1)	0.04^*∗*^
Name of the village						
Makuyuni	81	12.3	1		1	
Meserani	79	12.7	1.1 (0.6–2.1)	0.4	1.5 (0.7–3.2)	0.3
Kimokouwa	64	43.1	3 (2.7–16.2)	0.001	3.1 (2.2–14.4)	0.001^*∗*^
Orbomba	86	43.7	3.2 (2.8–13.5)	0.001	3.4 (2.5–16.4)	0.001^*∗*^

^*∗*^ *p* < 0.05, missing value. Age of the mother from 16–19 years (*n* = 9). COR: crude odds ratio; AOR: adjusted odds ratio; CI: confidence intervals.

## Additional Points

*Study Limitations*. The cross-sectional design of the study could not allow for determination of a cause-effect relationship between diarrhea associated infections and WASH. Also some of the information obtained through questionnaire relied on mothers' self-reporting which might have involved recall bias. The findings cannot be generalized to all pastoral communities because the study was conducted in semipastoral communities setting and with a limited sample size.
